# Combining Experimental Assays and Molecular Modeling to Evaluate Monosubstituted Cinnamic Acid Derivatives as PDE4B Inhibitors

**DOI:** 10.1021/acsomega.5c07879

**Published:** 2025-12-10

**Authors:** Dongsheng Zhao, Wendi Jia, Wanyu Gong, Lingling Zhang, Zining Cui

**Affiliations:** † Department of Pharmacy, 442537 Quanzhou Medical College, Quanzhou 362000, China; ‡ State Key Laboratory of Green Pesticide, Integrative Microbiology Research Centre, Guangdong Province Key Laboratory of Microbial Signals and Disease Control, College of Plant Protection, 12526 South China Agricultural University, Guangzhou 510642, China

## Abstract

In this study, we evaluated the inhibitory effects of cinnamic acid and its 18 commercially available derivatives on phosphodiesterase 4 (PDE4) to investigate their therapeutic potential for chronic obstructive pulmonary disease (COPD). Among the tested compounds, *p*-coumaric acid and *trans*-4-methoxycinnamic acid exhibited potent PDE4B inhibitory activity (IC_50_ = 2.2 μM and 8.2 μM, respectively) and notable selectivity over PDE4D, with IC_50_ values against TNF-α release in human mononuclear cells of 21.5 μM and 30.8 μM, respectively, comparable to rolipram. Structure–activity relationship (SAR) analysis indicated that *para*-substituted derivatives generally showed higher activity than their *meta*- or *ortho*-substituted counterparts. A validated CoMSIA model (*q* ^2^ = 0.514, *r* ^2^ = 0.971) highlighted the importance of electrostatic properties, revealing that electron-donating groups at the *para* position enhance inhibitory activity. Molecular docking illustrated that active derivatives bind in the PDE4B active site, forming key interactions with Gln443 and His234, which was refined by molecular dynamics simulations and free energy calculations. For *p*-coumaric acid, binding is primarily driven by a strong hydrogen bond with His234, whereas for *trans*-4-methoxycinnamic acid, enhanced hydrophobic interactions within the M pocket compensate for the lack of this hydrogen bond, revealing a dual mechanism for high-affinity binding. *In vivo* studies further confirmed significant anti-inflammatory effects, where *p*-coumaric acid inhibited TNF-α release by 41.1% and LPS-induced neutrophilia by 32.5%. Additionally, in silico ADMET profiling predicted favorable drug-like properties including high oral bioavailability and low CNS penetration, while identifying CYP2C8 inhibition as an optimizable liability. These integrated results underscore monosubstituted cinnamic acids, especially *para*-hydroxy and *para*-methoxy derivatives, as privileged scaffolds for developing novel PDE4B-targeted therapeutics for COPD.

## Graphical abstract

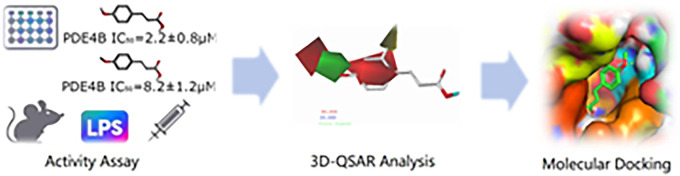

## 1 Introduction

Inflammation is one of the most common pathological processes in human diseases, and various inflammatory reactions are related to the concentration of intracellular second messenger cyclic adenosine monophosphate (cAMP).^1–4^ Increasing the intracellular cAMP level can inhibit the effects of various cells, such as lymphocytes, macrophages, neutrophils, endodermal cells and lung epithelial cells. The intracellular concentration of cAMP is regulated by both the rate of cAMP synthesis by receptor-linked adenylyl cyclase and the rate of cAMP degradation by phosphodiesterase 4 (PDE4) enzyme.^5,6^ Therefore, PDE4 enzyme inhibitors can increase the intracellular cAMP concentration, thus playing a role in inhibiting inflammation.^7^

For a quite long time, researchers have developed a variety of PDE4 inhibitors, among which roflumilast is used to treat chronic obstructive pneumonia;^8^ rolipram was not marketed due to its serious adverse reactions (such as dizziness, headache, nausea, vomiting, etc.), and was usually used as a positive control drug in subsequent studies of PDE4 inhibitors.^9–11^ Cilomilast was also discontinued due to side effects and other reasons.^12^ 3,4,5-Trihydroxycinnamic acid (THCA) has been reported to have anti-inflammatory and antioxidant activities, and oral administration of THCA in model animals significantly inhibited tumor necrosis factor-α (TNF-α) in bronchoalveolar lavage fluid of experimental COPD mice. It can inhibit the expression of phosphodiesterase 4 (PDE4) in pulmonary inflammatory cells of experimental COPD mice.^13^

To investigate the PDE4 inhibitory activity of monosubstituted cinnamic acid derivatives and their therapeutic potential for COPD, we quantitatively evaluated the inhibitory effects of cinnamic acid and 18 commercially available derivatives against PDE4B and PDE4D. Our integrated approach combined experimental and computational techniques: after identifying leads with potency comparable to rolipram, we developed a robust 3D-QSAR (CoMSIA) model to define the key steric and electrostatic features for activity, employed molecular docking to hypothesize binding modes, and utilized molecular dynamics simulations to elucidate the dynamic binding mechanism and critical interactions stabilizing the inhibitor-PDE4B complex. This multifaceted strategy aims not only to identify potent new chemotypes but also to provide a solid structural foundation for the rational design of next-generation PDE4B inhibitors.

## 2 Materials and Methods

### 2.1 Activity Assay

#### 2.1.1 Assay of Human PDE4 Activity

The inhibitory activities of commercial cinnamic acid derivatives (purchased from Sigma-Aldrich; identity and purity (≥95%) confirmed by Certificate of Analysis) against PDE4 were evaluated. A standard procedure for PDE testing was carried out according to the previously mentioned protocol.^5,14^ The enzyme was prepared from U937 cells which were derived from human monocytes, and was stored at −20 °C after preparation. Measurement of PDE4 activity was performed using this stored enzyme after it was diluted with distilled water containing bovine serum albumin. The substrate solution was prepared by adding [3H]-cAMP (300,000 dpm (5000 Bq)/ assay) and 100 mmol/L cAMP solution to 100 mmol/L Tris-HCl (pH 8.0) containing 5 mmol/L ethylene glycol-bis (β-aminoethyl ether) and *O*,*O*′-bis­(2-aminoethyl)­ethylene glycol-*N*,*N*,*N*′,*N*′-tetraacetic acid. The substrate solution was mixed with the enzyme solution containing a test compound dissolved in DMSO, and incubation was done for 30 min at 30 °C. The solvent (DMSO) alone was used as a negative control and showed no inhibitory activity against PDE4B at the highest concentration tested, confirming the assay validity.Assays were performed in duplicate at different concentrations of each test compound.

#### 2.1.2 Assay of TNF-α Release

The blood was mixed with saline at a ratio of 1:1, and the peripheral blood mononuclear cells (PBMCs) were isolated from buffy coats using Lymphoprep tubes.^15^ The PBMCs were suspended in RPMI 1640 with 0.5% human serum albumin, pen/strep, and 2 mM l-glutamine at 5 × 10^5^ cells/mL. The cells were preincubated with the test compounds in 96-well plates for 30 min and stimulated for 18 h with 1 mg/mL lipopolysaccharide. TNF-α concentration in the supernatants was measured by homogeneous time-resolved fluorescence resonance (TR-FRET). The assay was quantified by measuring fluorescence at 665 nm (proportional to TNF-α concentration) and 620 nm (control). Results are expressed as IC_50_ values (μM).

#### 2.1.3 LPS Induced Sepsis for Measurement of TNF-α Inhibition in Mice

The sepsis model in mice was induced using lipopolysaccharide (LPS) according to the methods described in the literature.^16^ Female Swiss albino mice were selected according to the body weights, which were equivalent within each group. The mice were fasted for 20 h with free access to water and dosed for oral administration (po) with the test compounds suspended in vehicle containing 0.5% Tween 80 in 0.25% sodium salt of carboxymethylcellulose. The control mice received the vehicle alone. After 30 min of oral dosing, the mice were injected intraperitoneally with 500 μg of lipopolysaccharide (*Escherichia coli*, LPS: B4 from Sigma) in phosphate buffer. Then the mice were bled via retro-orbital sinus puncture after 90 min of LPS administration. Serum samples were collected by centrifuging the blood samples at 4000 rpm for 20 min, which were stored overnight at 4 °C. The serum samples were then immediately checked for TNF-α levels using commercial mouse TNF-α ELISA kit (Amersham Biosciences) and assay was carried out following the manufacturer’s instruction.

#### 2.1.4 LPS Induced Neutrophilia Model for Asthma and COPD

Neutrophilia was induced in Sprague–Dawley rats by lipopolysaccharide (LPS) following the established protocol.^15^ Male Sprague–Dawley rats were acclimatized to laboratory conditions for 1 week prior to the experiment. According to the body weight, the rats were distributed to various groups randomly. Except for the normal group, all the rats were exposed to 100 μg/mL lipopolysaccharide (*E. coli*, LPS: B4 from Sigma) for 40 min. The rats were dosed with the test compound suspended in the vehicle containing 0.25% carboxymethyl cellulose before half an hour of LPS exposure. Bronchoalveolar lavage (BAL) was performed 6 h after LPS exposure, total cell count and differential leukocyte count (DLC) were performed and compared with control and the standard drug.

### 2.2 3D-QSAR Analysis

The 3D-QSAR analysis was conducted using the CoMSIA module of SYBYL-x2.0 ^17^ software installed on a DELL T3630 workstation. The 19 tested compounds were divided into a training set and a test set based on their chemical structural characteristics and activity gradient, with 14 compounds in the training set and 5 compounds in the test set (see in Table 1). The IC_50_ values of all compounds for PDE4B inhibition activity were converted into log­(1/IC_50_) (denoted as pIC_50_), which can be directly used by the software for modeling. The three-dimensional structures of all compounds were assigned the MMFF94 force field and MMFF94 charges, and energy optimization was performed using the Powell gradient algorithm. The maximum number of iterations was set to 10,000, and the iteration converged when the energy difference was no greater than 0.005 kcal·mol^–1^. The compounds were aligned using cinnamic acid as the template molecule. After calculating the CoMSIA fields for each compound, a statistical model was established using Partial Least Squares regression with pIC_50_ values. The maximum number of principal components (N) and the validation correlation coefficient (*q* ^2^) were obtained through Leave-One-Out validation. After determining the optimal number of principal components, a no-validation model was constructed under this principal component, and the statistical parameters of the model were obtained. The constructed model was used to predict the activity of the 14 compounds in the training set and the 5 compounds in the test set, thereby evaluating the model quality.

**Table 1:**
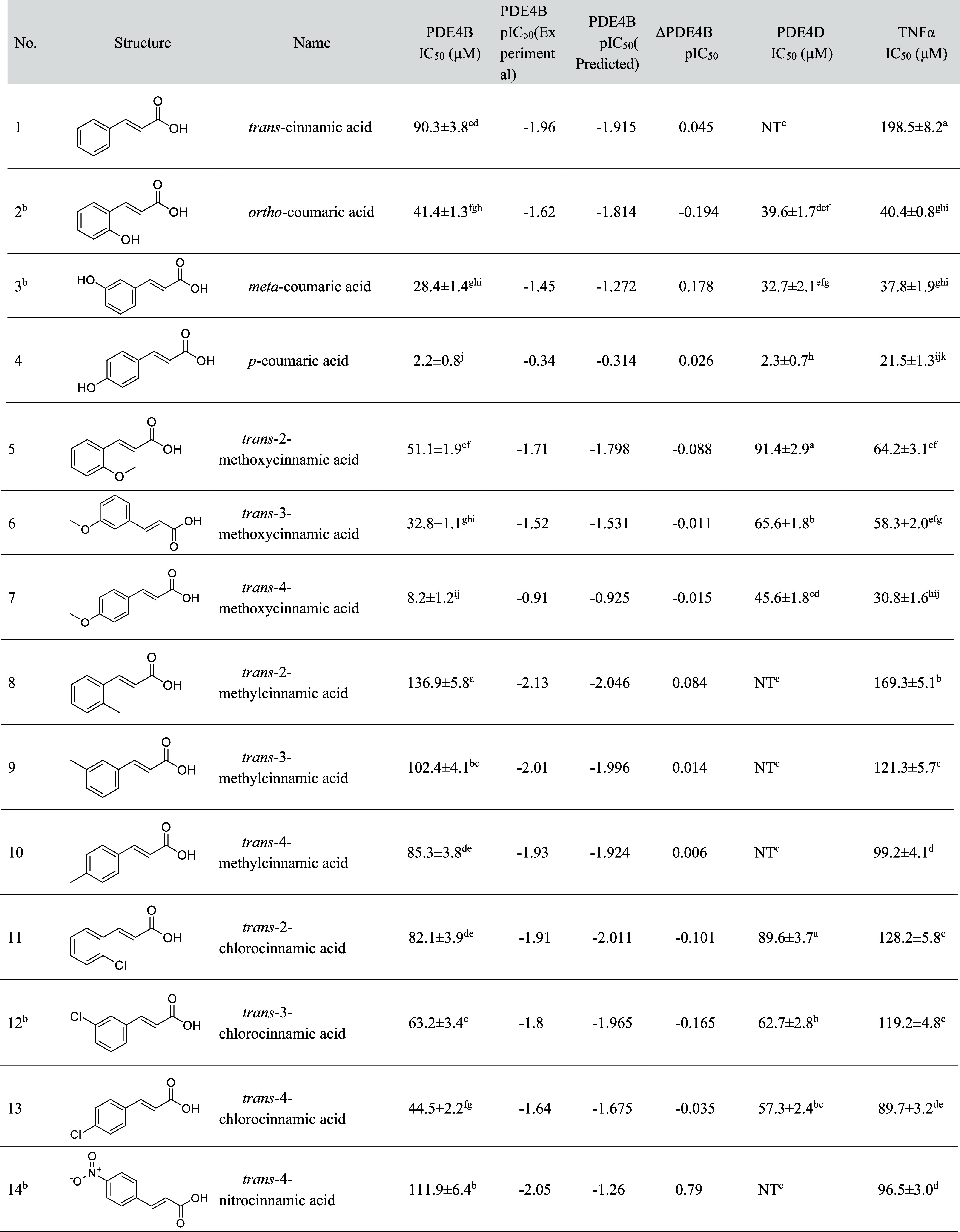

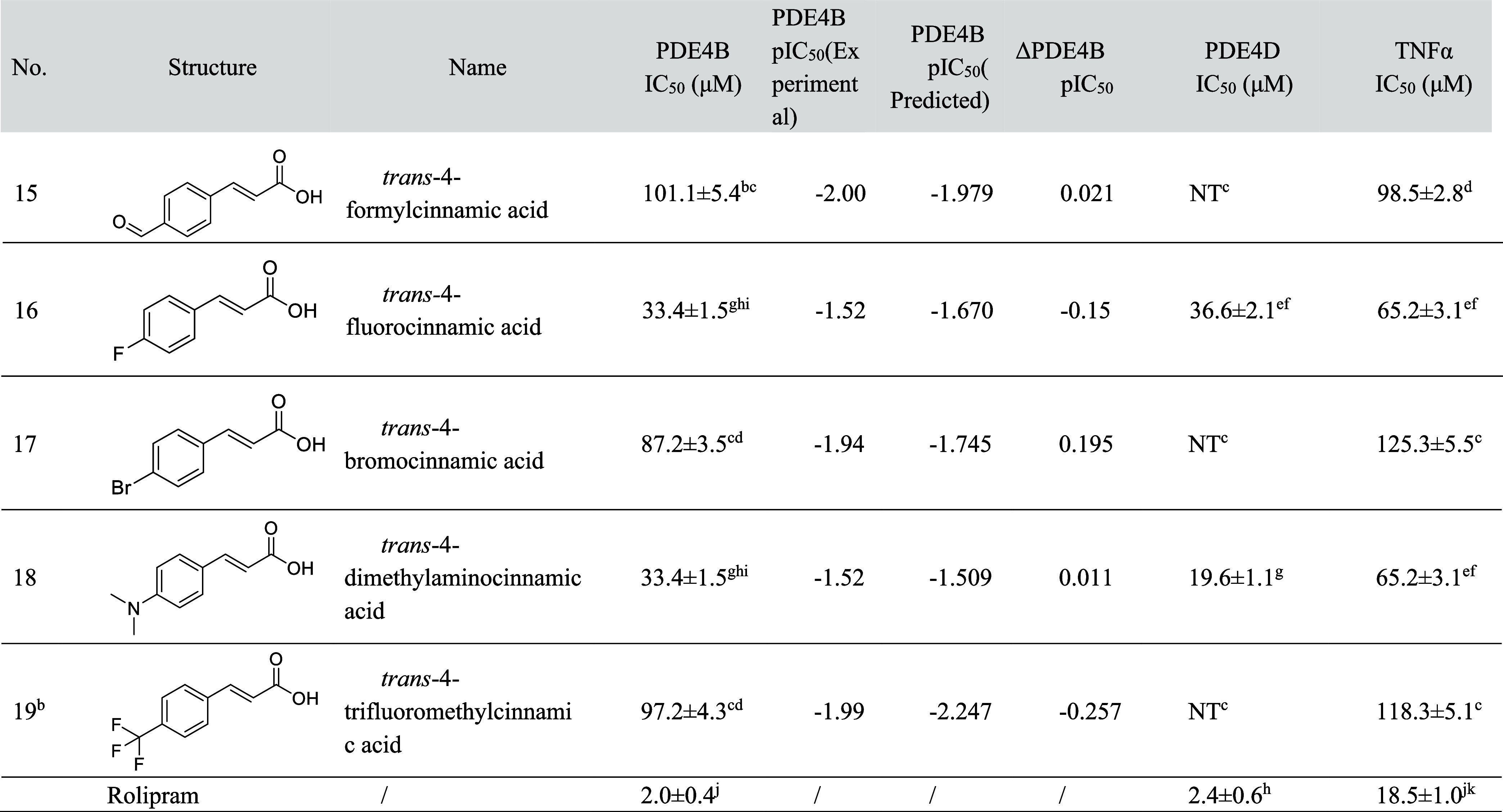
Inhibitory Activities of Cinnamic Acid Derivatives Against PDE4 and TNF-α Release, with CoMSIA Model Predictions^a^,^d^

a Results are the average of at least three assays.

b Testset compounds in CoMSIA modeling.

c NT, not tested.

d The letters a-k denoted the results of difference significance analysis. Means followed by the same letter within the same column are not significantly different (*p* ≥ 0.05, Fisher’s LSD multiple comparison test).

### 2.3 Molecular Docking

#### 2.3.1 Docking Parameters and Protocol Validation

All molecular docking studies were performed using AutoDock 4.2 ^18^ software installed on a DELL T3640 workstation. The docking protocol was rigorously validated by redocking and cross-docking. For redocking, the native ligand (rolipram) was extracted from the PDE4B-rolipram complex (PDB ID: 1XN0)^19^ and all water molecules were removed. A grid box of 70 × 70 × 70 points (spacing of 0.375 Å) was centered on the N atom of the key residue Gln443 to encompass the entire binding site. Twenty independent docking runs were performed using the Lamarckian Genetic Algorithm (LGA) with 50 searches per run, each initiated with a different random seed. A successful pose was defined as having a root-mean-square deviation (RMSD) of heavy atoms ≤ 2.0 Å from the crystallographic pose.

#### 2.3.2 Cross-Docking Validation

To further assess the robustness and transferability of the established protocol, a cross-docking study was performed using ten additional PDE4B crystal structures, whose crystallographic parameters are provided in Supporting Table S1. These structures encompass both wild-type and mutant enzymes, cocrystallized with ligands of diverse chemotypes. A critical step was to define a consistent grid box center across all structurally aligned complexes. This was achieved by programmatically aligning the A-chain of each target structure to the reference structure (PDB: 1XN0) using PyMol.^20^ The coordinates of the N atom of Gln443 from 1XN0 were then transformed into the framework of each target structure to serve as the unbiased grid center. The specific grid center coordinates determined for each complex are provided in Supporting Table S2. All other docking parameters remained identical to those used in the redocking validation.

#### 2.3.3 Molecular Docking of Cinnamic Acid Derivatives

Following the validation of the docking protocol in both redocking and cross-docking scenarios, molecular docking studies were performed to investigate the binding interactions of cinnamic acid derivatives with the wild-type PDE4B (PDB: 1XN0). After docking, the resulting poses were subjected to cluster analysis based on a histogram of conformational populations. The representative pose with the lowest binding energy that also aligned with the key chemical features of the rolipram-PDE4B (PDB ID: 1XN0) was selected from the largest conformational cluster. Protein–ligand interactions were analyzed and visualized three-dimensionally using Discovery Studio Visualizer.^21^

To further evaluate the binding efficiency of the ligands, the Ligand Efficiency (LE) metric was calculated for each derivative using the following equation

LE=−ΔG/N

where Δ*G* represents the predicted binding free energy (in kcal·mol^–1^) and *N* is the number of non-hydrogen atoms in the ligand. LE provides an estimate of the binding energy per heavy atom, aiding in the assessment of a compound’s optimization potential and facilitating comparisons across ligands of different sizes.

### 2.4 Molecular Dynamics (MD) Simulation

Molecular dynamics simulations were performed using the GROMACS 2023.4 software package.^22^ The CHARMM36 force field^23^ was applied for the protein, and the ligand parameters for 4-hydroxycinnamic acid were generated using the CGenFF program.^24^ The complex was solvated in a cubic box with TIP3P water molecules. The system’s temperature and pressure were maintained at 303.15 K and 1 bar, respectively, using the v-rescale thermostat and the Parrinello–Rahman barostat. The Particle Mesh Ewald (PME) method was employed to handle long-range electrostatic interactions, with a cutoff of 1.2 nm set for both Coulombic and van der Waals interactions. The simulation utilized a 2 fs integration time step.

The system underwent a standard equilibration protocol prior to the production run. First, energy minimization was conducted using the steepest descent algorithm^25^ to relieve any steric clashes. This was followed by a 100 ps equilibration in the NVT (canonical) ensemble to stabilize the temperature, and subsequently a 100 ps equilibration in the NPT (isothermal–isobaric) ensemble to adjust the density. Finally, a production MD simulation was carried out for 100 ns.

Trajectory analysis, including the calculation of the root-mean-square deviation (RMSD), root-mean-square fluctuation (RMSF), radius of gyration (*R*_g_), and solvent-accessible surface area (SASA), was performed using the built-in tools of GROMACS. The binding free energy was calculated using the MM/PBSA approach as implemented in the gmx_MMPBSA tool.

### 2.5 *In Silico* ADMET Prediction

The *in silico* absorption, distribution, metabolism, excretion, and toxicity (ADMET) properties of the key bioactive compounds, *p*-coumaric acid and *trans*-4-methoxycinnamic acid, were predicted using the ADMETlab 3.0 platform (https://admetlab3.scbdd.com/).^26,27^ The canonical SMILES notations of the compounds served as the input for the analysis. A comprehensive set of parameters was evaluated to profile the drug-likeness and potential liabilities of these leads. This included key end points such as passive membrane permeability (PAMPA), P-glycoprotein interaction, oral bioavailability (F20%), blood-brain barrier penetration (BBB), plasma protein binding (PPB), inhibition and substrate potential for critical cytochrome P450 enzymes (e.g., CYP3A4, CYP2C8), plasma half-life (*T*_1/2_), and major toxicity concerns including human hepatotoxicity, hERG channel inhibition, skin sensitization, eye irritation, and mutagenicity (AMES test). All predictions were conducted using the platform’s integrated algorithms and default settings.

## 3 Results and Discussion

### 3.1 Activity Assay

#### 3.1.1 Assay of Human PDE4 Activity

The *in vitro* inhibitory effects of the 19 compounds against PDE4B and PDE4D were assessed using the previously described enzymatic assay, with rolipram serving as the positive control. Data for their ability to inhibit the release of TNF-α in human blood mononuclear cells (HM) was also reported. The IC_50_ (the half maximal inhibitory concentration) values are shown in Table 1. The results revealed that the cinnamic acid and its derivatives exhibit good inhibitory effects on PDE4B and PDE4D. Among them, the activity of *p*-coumaric acid and *trans*-4-methoxycinnamic acid is significantly higher than that of cinnamic acid. Based on the activity data of coumaric acid, *trans*-4-methoxycinnamic acid, methylcinnamic acid, and chlorocinnamic acid, it is found that compounds with *para*-substitution have higher activity than those with *meta*-substitution, and compounds with *meta*-substitution have higher activity than those with *ortho*-substitution. Especially, *p*-coumaric acid and *trans*-4-methoxycinnamic acid have inhibitory effects comparable to the positive control drug rolipram. *Trans*-4-methoxycinnamic acid slightly decreased inhibitory activity against PDE4B but resulted in the remarkable loss of inhibitory activity against PDE4D, and exhibited higher selectivity ratios for PDE4B over PDE4D than rolipram. The observed selectivity of *trans*-4-methoxycinnamic acid may stem from subtle differences in the hydrophobic clamp region and the M-loop flexibility between the two subtypes. The *para*-methoxy group might be optimally accommodated in the PDE4B binding pocket, while experiencing steric or electronic incompatibility in the PDE4D active site.

#### 3.1.2 Assay of TNF-a Release

The inhibitory effect of the 19 compounds on the release of HM-TNF-α correlated well with their potency in inhibiting PDE4. In the HM-TNF-α assay, most of the compounds showed slightly lower potency compared to rolipram, with the exception of *p*-coumaric acid and *trans*-4-methoxycinnamic acid. These two compounds exhibited IC_50_ values of 21.5 μM and 30.8 μM, respectively, which were comparable to the potency of rolipram in this assay. This suggests that *p*-coumaric acid and *trans*-4-methoxycinnamic acid have additional beneficial effects on the inhibition of TNF-α release in human blood mononuclear cells through PDE4 inhibition. The well-established correlation between PDE4 inhibition and TNF-α suppression is mechanistically explained by the following pathway: By inhibiting PDE4, these compounds elevate intracellular cAMP levels, which in turn activates protein kinase A (PKA). PKA phosphorylates and inhibits key transcription factors like NF-κB, leading to the downregulation of pro-inflammatory cytokines such as TNF-α. This mechanism provides a solid foundation for understanding the anti-inflammatory effects observed in our study.

#### 3.1.3 LPS Induced TNF-α in SA Mice and Neutrophil Influx in BALF of SD Rats

*In vivo* experiments were conducted using an LPS-induced sepsis model to measure TNF-α inhibition in female Swiss Albino mice and to assess neutrophilia inhibition for asthma and COPD in male Sprague–Dawley rats. Details such as the oral dosages and the number of animals used, along with the percentage inhibition of neutrophilia calculated from the DLC, are provided in Table 2. The findings revealed that *p*-coumaric acid demonstrated significant inhibitory effects on TNF-α release (41.1%) and LPS-induced neutrophilia (32.5%), which were comparable to the positive control rolipram (41.7% and 32.8%). In contrast, the inhibitory effects of *trans*-4-methoxycinnamic acid (29.6% and 24.2%) were slightly lower than those of rolipram. These findings are particularly relevant in the context of COPD pathophysiology, where both TNF-α-driven inflammation and neutrophilic influx are well-established key drivers of tissue damage and disease progression.

**Table 2:** LPS Induced TNF-α in SA Mice and Neutrophil Influx in BALF of SD Rats

	Swiss Albino mice (*n* = 6)		Sprague–Dawley rats (*n* = 6)	
compds.	does (mg/kg, po)	TNF-α inhibition (%)	does (mg/kg, po)	LPS induced neutrophilia (% inhibition)
*p*-coumaric acid	10	41.1 ± 0.65	10	32.5 ± 0.33
*trans*-4-methoxycinnamic acid	10	29.6 ± 0.36	10	24.2 ± 0.52
rolipram	10	41.7 ± 0.78	10	32.8 ± 0.40

It is important to note that the LPS-induced acute inflammation models employed here, while well-established for initial proof-of-concept screening, do not fully capture the chronic and complex pathophysiology of human COPD. Nonetheless, these models serve as a valid and reproducible platform in early drug discovery to demonstrate fundamental *in vivo* anti-inflammatory efficacy and pharmacokinetic suitability. The significant bioactivity observed for our lead compounds justifies their further investigation in more complex and clinically relevant chronic models, such as those involving long-term cigarette smoke exposure.

### 3.2 3D-QSAR Analysis

#### 3.2.1 CoMSIA Model Construction and Validation

Based on a training set of 14 compounds, a CoMSIA model was developed. The key statistical parameters of the model, including the optimal number of principal components (*N*), the cross-validated correlation coefficient (*q* ^2^), the conventional correlation coefficient (*r* ^2^), the standard error of estimation (SEE), and the F-test value, are summarized in Table 3.

**Table 3:** Statistical Parameters of the CoMSIA Model

	statistical parameters					fraction (%)			
model	*N*	*q* ^2^	*r* ^2^	*S*	*F*	steric	donor + acceptor	electrostatic	hydrophobic
CoMSIA	3	0.514	0.971	0.095	111.882	4.4	38.9	42.7	14

The model demonstrated excellent performance on the training set, indicated by a high noncross-validated correlation coefficient (*R* ^2^ = 0.971, *N* = 3) and a low standard error of estimate (SEE = 0.095). This indicates a strong capability for capturing the structure–activity relationships within the training data. While the leave-one-out cross-validated coefficient (*Q* ^2^ = 0.514) slightly exceeds the generally accepted threshold of 0.5, The model’s internal robustness and potential overfitting are validated by a subsequent test set.

The predictive ability of the CoMSIA model was further rigorously assessed using an external test set of five compounds. The initial predictive correlation coefficient for the entire test set was low (*R* ^2^_pred = 0.08, *n* = 5). This poor overall performance was primarily due to one significant outlier, *trans*-4-nitrocinnamic acid. The inability of the model to predict this compound’s activity is not necessarily an indication of poor model robustness, but rather a consequence of its unique structural features. From an electrostatic perspective, the nitro group possesses an exceptionally strong electron-withdrawing capacity, imparting a highly positive electrostatic potential to the benzene π-system through both inductive and conjugative effects. The influence of substituents in all other compounds (in both the training and test sets) on the benzene ring’s electrostatic potential is markedly weaker. Sterically, the planar geometry of the nitro group also contrasts significantly with the three-dimensional bulk of substituents found in other compounds.

After excluding this outlier, the model demonstrated outstanding predictive power for the remaining four compounds in the external test set (*R* ^2^_pred = 0.93). A scatter plot of the experimental versus predicted activity values for both the training and test sets is provided in Figure 1A,B, while a residual plot of the experimental versus predicted activity values is displayed in Figure 1C. The combination of a high *R* ^2^_pred value with a moderate *Q* ^2^ value indicates that while the model may be somewhat sensitive to variations in the training set composition, the fundamental structure–activity relationships it has identified possess significant predictive value for new compounds with analogous structures. These insights will be instrumental in guiding further structural optimization efforts.

**Figure 1:**
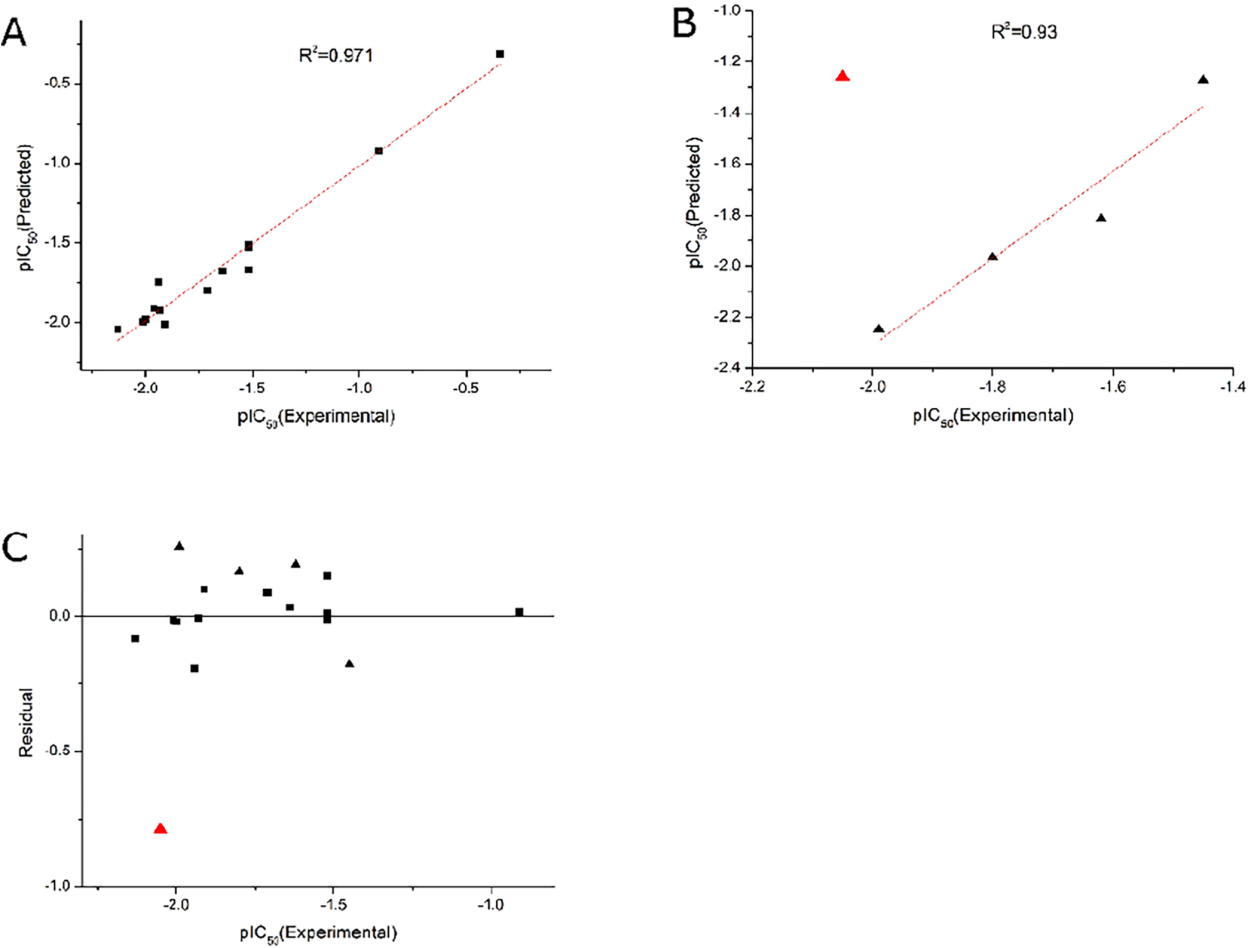
Correlation plots of predicted versus experimental values for the training set ((A), *R* ^2^ = 0.971, *n* = 14) and test set (B), *R* ^2^ = 0.93, *n* = 5) compounds derived from the CoMSIA model. The residual plot for all compounds is shown in ((C), *n* = 19). Filled black squares: training set compounds; filled black triangles: test set compounds; filled red triangles: outlier.

#### 3.2.2 CoMSIA Model Analysis

The CoMSIA model provided visualized steric and electrostatic field contour maps, with high-, medium-, and low-activity compounds selected as representative examples for analysis. As shown in Figure 2A–C, green contours indicate sterically favorable regions where bulky substitutions enhance activity, as exemplified by the highly active *p*-coumaric acid. Conversely, yellow contours mark sterically disfavored regions that reduce activity, such as in *trans*-2-methylcinnamic acid. Although *trans*-2-methoxycinnamic acid does not align with the steric field preferences, it exhibits moderate activity due to favorable electrostatic interactions. All highly active derivatives among the 19 compounds were *para*-substituted, consistent with the steric constraints of the model. However, *trans*-4-formylcinnamic acid and *trans*-4-trifluoromethylcinnamic acid showed unexpectedly low activity.

**Figure 2:**
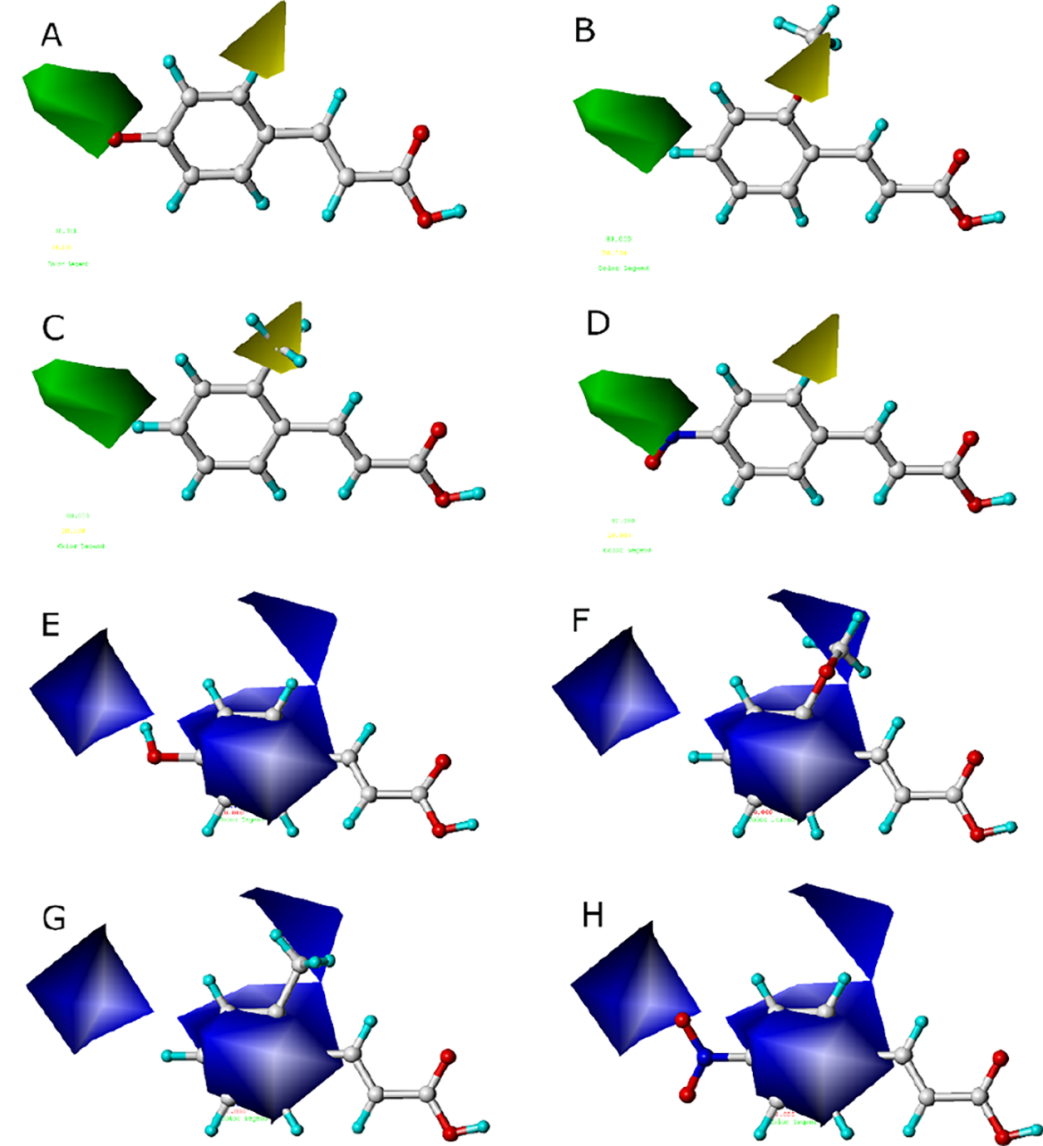
CoMSIA results. (A–D): Steric field correspondence of *p*-coumaric acid, *trans*-2-methoxycinnamic acid, *trans*-2-methylcinnamic acid and *trans*-4-nitrocinnamic acid. Green contours: sterically favorable regions that enhance activity; yellow contours: sterically unfavorable regions that reduce activity. (E–H): Electrostatic field correspondence of the same compounds. Blue contours: regions where electronegative groups improve activity.

Analysis of the electrostatic field (Figure 2E–G) revealed that substituents with high electron density or those capable of increasing electron density on the benzene ring-such as in *p*-coumaric acid and *trans*-4-methoxycinnamic acid-correspond to blue contours, indicating that electronegative groups favor enhanced activity. The absence of red contours (electropositive-favorable regions) reflects the lack of corresponding substituents among the compounds studied.

Notably, the outlier *trans*-4-nitrocinnamic acid, despite being *para*-substituted, was poorly predicted by the model. Its nitro group introduces an exceptionally strong electron-withdrawing effect and a planar steric profile that diverge significantly from the electronic and spatial features captured in the contour maps, explaining its deviation from the predicted activity. The matching of its contour map with the CoMSIA model is shown in Figure 2D,H.

The model also included hydrogen bond donor and acceptor fields; however, detailed analysis of these features was omitted, as hydrogen-bond capable groups were generally marked as favorable. In summary, the CoMSIA model suggests that *para*-substitutions with high electron-density groups or hydrogen-bond forming moieties are most likely to yield highly active cinnamic acid derivatives.

### 3.3 Molecular Docking

#### 3.3.1 Validation of the Docking Protocol

The reliability of the molecular docking workflow was first assessed by redocking the cocrystallized ligand (rolipram) into its native structure (1XN0). The protocol successfully reproduced the native pose across 20 independent runs, yielding a low mean RMSD of 1.34 ± 0.33 Å and a consistent lowest binding energy of −8.00 ± 0.04 kcal/mol (Supporting Table S3). The accuracy and reproducibility of the redocking procedure are shown in Figure 3A (a single, representative redock superimposed on the native pose) and Supporting Figure S1 (superposition of all 20 independent runs). Clustering analysis revealed a frequent convergence to a dominant, low-energy binding mode. Although nuances such as near-isoenergetic clusters and variability in cluster size (19–43 members) were observed, these reflect the inherent stochasticity and conformational flexibility of the system, rather than undermining the protocol’s robustness.

**Figure 3:**
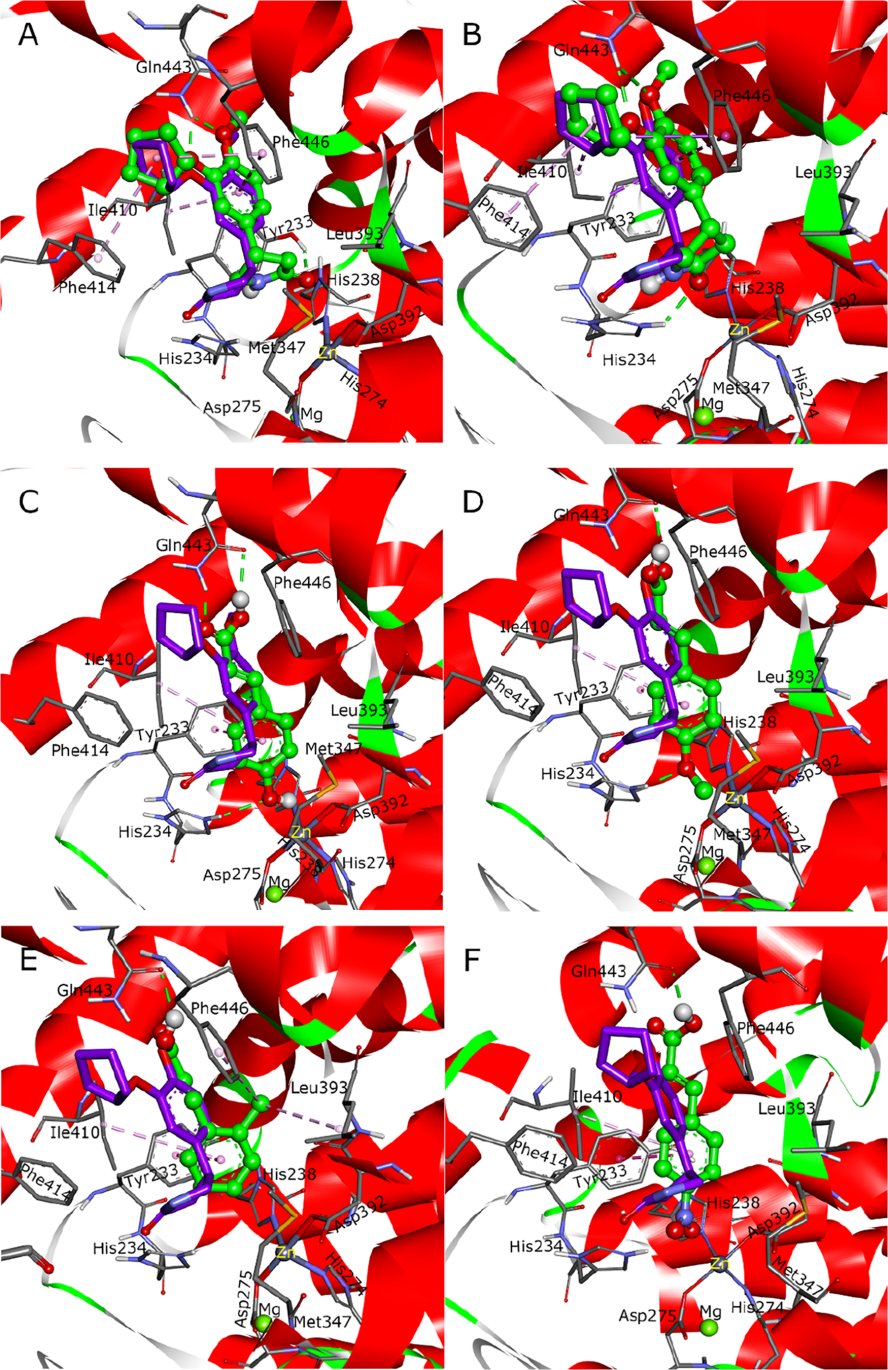
Molecular docking results. The purple sticks represent the crystallographic conformation of rolipram in PDE4B (PDB: 1XN0), and the green ball and sticks represent the docked pose. Green dashed lines denote hydrogen bonds, and light pink dashed line represent π–π and π-alkyl interactions. (A) Redocking result of rolipram. (B) Cross-docking result of rolipram from the wild-type PDE4B (1XN0) into the mutated PDE4B (3O56). Predicted binding poses of (C) *p*-coumaric acid, (D) *trans*-4-methoxycinnamic acid, (E) *trans*-2-methylcinnamic acid, and (F) *trans*-4-nitrocinnamic acid.

The protocol’s transferability was further evaluated by cross-docking against ten diverse PDE4B structures. The results demonstrated strong generalizability, with a mean RMSD of 1.06 ± 0.37 Å and a mean binding energy of −8.37 ± 0.44 kcal/mol (Supporting Table S3). The conservation of the core binding motif in the most accurate prediction and the structural alignment of the other nine PDE4B binding sites are illustrated in Figure 3B and Supporting Figure S2, respectively. In one instance (1XM6), a favorable energy (−9.34 kcal/mol) coincided with a higher RMSD (1.45 Å). This can be attributed to scoring function limitations or binding site flexibility allowing for distinct yet plausible orientations. Such anomalies highlight the complexity of molecular recognition but do not detract from the overall validity of the protocol, which reliably identifies potent binding modes across diverse ligand-protein complexes.

During the cross-docking validation, the predicted pose of rolipram positioned the carbonyl oxygen atom of its pyrrolidine ring in close proximity to the catalytic Zn^2+^ ion, suggesting a potential electrostatic interaction or metal coordination. It is important to emphasize that the standard scoring function of AutoDock does not explicitly parametrize metal coordination bonds; therefore, this result should be interpreted only as the identification of a favorable interaction region rather than the confirmation of a specific coordination geometry.

#### 3.3.2 The Docking Study of the Cinnamic Acid Derivatives

Molecular docking was employed primarily to elucidate the potential binding modes and rationalize the structure–activity relationships (SAR) of the cinnamic acid derivatives, rather than to provide quantitative binding affinity predictions. While this approach successfully provided valuable insights, its inherent limitations must be considered. The accuracy of absolute binding energy predictions is constrained by current scoring functions, which struggle to fully account for conformational flexibility, entropic contributions, and explicit solvent effects. This is evidenced by the clustering of predicted binding energies around −4.0 kcal/mol (Supporting Table S4), corresponding to millimolar-range *K*_i_ values that deviate from the experimental micromolar IC_50_ values-a known discrepancy for small, flexible ligands. It should be noted that this approach provides a static view of protein–ligand interactions; consequently, our analysis focused on the relative orientations and key interaction patterns of the docked poses.

For each compound, the representative pose from the largest and lowest-energy cluster was selected for detailed analysis. As shown in Figure 3C–F, the high-affinity ligands *p*-coumaric acid and *trans*-4-methoxycinnamic acid are well accommodated within the PDE4B binding pocket. Their carboxyl groups form a key hydrogen bond with Gln443, while their *para*-substituents (hydroxy and methoxy groups) engage in an additional hydrogen bond with the NH of His234. Critically, the poses of these active ligands also revealed optimal engagement with key hydrophobic regions: the planar aromatic ring was positioned to engage in π–π and π-alkyl interactions with residues Ile410 and Phe446 of the conserved “P-clamp”, while simultaneously forming hydrophobic contacts with Met347, a residue lining the metal-binding pocket (M pocket). This orientation allows the ligand to bridge both the P-clamp and the M pocket effectively. This predicted binding mode, which simultaneously engages the P-clamp and forms a hydrogen bond with His234 in the M pocket, represents a hallmark of potent PDE4 inhibition.^9,19^ In contrast, this optimal dual-site engagement was absent in the poses of less active compounds, such as *trans*-2-methylcinnamic acid and *trans*-4-nitrocinnamic acid. The strong correlation between these predicted interaction patterns and the experimental activity trends provides a plausible structural rationale for the observed SAR.

Furthermore, evaluation of Ligand Efficiency (LE) confirmed the high quality of the cinnamic acid scaffold. As shown in Supporting Table S4, the consistent LE values across most derivatives, including less active examples like *trans*-2-methylcinnamic acid (LE = −0.38), indicate that the core structure itself engages the PDE4B active site with good atomic economy. The low potency of these compounds likely stems from factors such as suboptimal substituent positioning or steric clashes.

This baseline efficiency highlights the exceptional profile of our lead compounds, *p*-coumaric acid and *trans*-4-methoxycinnamic acid. They distinguish themselves by marrying high potency (IC_50_ = 2.2 and 8.2 μM) with maintained ligand efficiency (LE ≈ −0.35), a hallmark of an optimizable lead series. The stark outlier, *trans*-4-nitrocinnamic acid (LE = −0.27), confirms that strong electron-withdrawing groups at the *para* position are detrimental, likely due to electronic incompatibility within the binding pocket.

### 3.4 Molecular Dynamics Simulations

Building upon the foundational binding hypotheses generated by molecular docking, we performed a 100 ns MD simulation on the complex of 4-hydroxycinnamic acid (*p*-coumaric acid) with PDE4B to evaluate the stability of the predicted pose and to obtain a dynamic view of the binding mechanism. The rapid equilibration of the system was confirmed by the stable RMSD (∼0.18 nm), RMSF (∼0.2 nm), Rg (∼1.98 nm), and SASA (∼158 nm^2^) of the protein throughout the production phase, as detailed in Supporting Figure S3. Furthermore, the free energy landscape (FEL) projected along the RMSD and *R*_g_, provided in Supporting Figure S3, featured a dominant basin within an RMSD of 0.16–0.20 nm and an *R*_g_ of 1.97–1.99 nm, confirming that the complex predominantly sampled a narrow, stable conformational ensemble. These results collectively confirm the formation of a stable protein–ligand complex. The MM/GBSA calculation yielded a binding free energy of −24.39 kcal/mol, affirming a strong binding affinity. The per-residue energy decomposition, presented in Figure 4A, revealed several key contributors. While residues coordinating the catalytic zinc ion (e.g., His238, His274, Asp275 and Asp392) exhibited substantial energy contributions-a hallmark of the conserved PDE4 active site-our analysis focuses on His234, Ile410, and Phe446, as they delineate the specific inhibitor-binding motif unique to the cinnamic acid scaffold. A representative snapshot from this equilibrated trajectory, captured at frame 64 (corresponding to 64 ns), is depicted in Figure 4B. However, the simulation revealed critical optimizations in the binding mode that underscore the limitations of static docking and provide a more dynamic, physiologically relevant mechanism.

**Figure 4:**
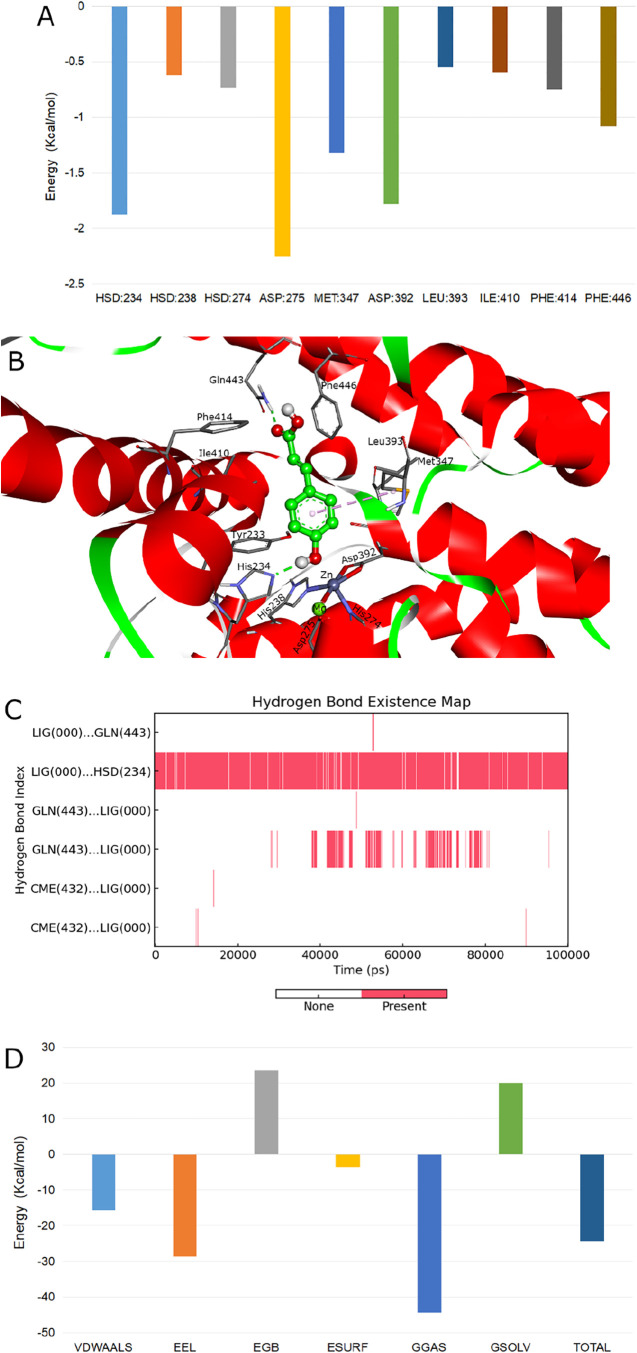
Molecular dynamics simulation results. (A) Decomposition of the binding free energy per residue. Key residues discussed in the text are highlighted. (B) Representative binding mode of the equilibrated complex, extracted from frame 64 (64 ns) of the MD trajectory, Green dashed lines denote hydrogen bonds, and light pink dashed line represents π-alkyl interaction. (C) Hydrogen bond occupancy between the ligand (LIG) and protein residues during the simulation. The donor and acceptor are shown on the left and right sides of the separator, respectively. CME denotes a nonstandard residue in the crystal structure. (D) Decomposition of the binding free energy by energy term. Abbreviations are VDWAALS, van der Waals energy; EEL, electrostatic energy in vacuum; EGB, electrostatic solvation energy (Generalized Born); ESURF, nonpolar solvation energy; GGAS, total gas-phase energy (EEL + VDWAALS); GSOLV, total solvation energy (EGB + ESURF); TOTAL, final estimated binding free energy.

Most notably, the hydrogen bonding network underwent a substantial redistribution. A critical refinement concerned the interaction with the invariant “Q-switch” residue Gln443. While the docking pose suggested a bidentate interaction, the simulation demonstrated that only the hydrogen bond from the amide hydrogen of Gln443 to the carbonyl oxygen of the ligand persisted with significant stability. The alternative hydrogen bond, involving the ligand’s carboxyl hydroxyl hydrogen, proved transient, consistent with the solvent-exposed nature of the Q pocket and the significant desolvation penalty, as reflected in its negligible net energy contribution. This observation, with its low overall occupancy of 18.98% visually represented in Figure 4C, aligns with the recognized role of Gln443 in directing ligand orientation rather than serving as a primary affinity anchor.^9,19^

In stark contrast, a fundamental optimization was revealed for the key hydrogen bond with His234. The static docking model predicted an interaction where the *para*-hydroxyl oxygen of the ligand acted as the acceptor for the hydrogen from His234 (N–H···O). In contrast, the equilibrated MD trajectory consistently showed a flipped, optimized configuration, with the ligand’s hydroxyl hydrogen acting as the donor to the nitrogen lone pair electrons of His234 (O–H···N). This flipped interaction is not only structurally distinct but also energetically superior, becoming exceptionally stable with an occupancy of 96.3%. This stark discrepancy is clearly reflected in the binding free energy decomposition presented in Figure 4C, where His234, located within the hydrophobic metal-binding pocket (M pocket), emerged as a major energy contributor (−1.8 kcal/mol), while Gln443 did not. This can be attributed to their distinct local environments: His234, buried in a hydrophobic region, pays a minimal desolvation penalty for hydrogen bond formation, resulting in a large net energy gain. Conversely, Gln443 in the solvent-exposed Q pocket incurs a significant desolvation cost that largely offsets the benefit of hydrogen bonding. Thus, the simulation identified His234, not Gln443, as the primary anchor point for the ligand’s polar interactions.

Furthermore, the hydrophobic interactions with the “P-clamp” were reinforced during the simulation. The distances observed in the docking pose suggested potential but suboptimal contacts with Ile410 and Phe446. The MD simulation revealed that these interactions tightened and became more persistent, as evidenced by the significant van der Waals contributions from these residues illustrated in the energy decomposition of Figure 4D. Concurrently, the favorable energies from Ile410 and Phe446 underscore the critical role of the hydrophobic ’P-clamp’ in stabilizing the ligand’s aromatic core. Notably, Met347, a key residue within the metal-binding pocket (M pocket), also contributed significantly to the binding affinity (∼−1.4 kcal/mol). The MD trajectory revealed that Met347 engages in stable hydrophobic interactions with the phenyl ring of the ligand, thereby enhancing the overall complementarity of the ligand within the M pocket. This observation reinforces the role of the M pocket not only as a site for specific polar interactions (via His234) but also as a region conducive to favorable hydrophobic contacts. This dynamic tightening allowed the planar aromatic ring of the inhibitor to be firmly sandwiched by the hydrophobic clamp, a hallmark of PDE4 inhibition that is consistently observed in structural studies^9,19^ and was fully validated by the dynamic simulation. These interactions with His234, Ile410, and Phe446 collectively define the characteristic binding mode of cinnamic acid derivatives within the PDE4B active site.

In summary, while the docking study provided a valid initial hypothesis, the MD simulation was crucial for elucidating the genuine binding mechanism. It demonstrated that the binding of 4-hydroxycinnamic acid is primarily driven by a strong, stable hydrogen bond with His234 in the M pocket and optimized van der Waals contacts with the P-clamp (Ile410 and Phe446). The additional hydrophobic stabilization provided by Met347 further consolidates the ligand’s position within the M pocket. This mechanism resonates with the established PDE inhibition paradigm, where the M pocket and hydrophobic clamp are key determinants of binding affinity.^9,19^ The role of Gln443 shifts from a primary hydrogen bond partner in the static model to a dynamic “steering” residue that contributes more to ligand orientation and specificity than to overall affinity. This refined mechanistic understanding, afforded by the integration of docking and dynamics, robustly supports the observed inhibitory activity of the compound.

### 3.5 Structure–Activity Relationship (SAR) Discussion

Integrating data from enzymatic assays (3.1), 3D-QSAR (3.2), molecular docking (3.3), and MD simulations (3.4), we have established a robust structure–activity relationship model for cinnamic acid derivatives.

The critical role of *para*-substitution for high activity is unequivocal, with *para*-substituted analogs exhibiting superior potency over *meta*- and *ortho*-substituted counterparts. Electron-donating or moderately electron-withdrawing groups at the *para* position are preferred, as consistently supported by CoMSIA steric and electrostatic contours. The binding mode refined by MD simulations provides the structural basis: a *para*-substitunt optimally accesses the deep, hydrophobic metal-binding pocket (M pocket).

Among *para*-substituted derivatives, the nature of the substituent fine-tunes potency. MD simulation of the most potent compound, *p*-coumaric acid (IC_50_ = 2.2 μM), revealed its phenolic hydroxyl group forms a highly stable hydrogen bond (96.3% occupancy) as a donor to the backbone of His234 in the M pocket, establishing it as a primary anchor. For *trans*-4-methoxycinnamic acid, which lacks a hydrogen bond donor, its high activity (IC_50_ = 8.2 μM) is maintained because the larger, more hydrophobic methoxy group compensates by forming enhanced van der Waals contacts with the hydrophobic M pocket environment (e.g., His234, Met347), coupled with a potentially lower desolvation penalty. This nuanced understanding—that the M pocket accommodates different *para*-substituents via distinct yet favorable mechanisms (hydrogen bonding vs enhanced hydrophobic packing)—greatly enriches our SAR model.

Stable engagement with the hydrophobic “P-clamp” (Ile410, Phe446) is essential for high affinity, as consistently observed. The role of Gln443, refined by MD, is primarily to steer ligand orientation via a stable, unidirectional hydrogen bond to the ligand’s carboxylate, rather than acting as a major affinity anchor.

In summary, the optimal binding motif for PDE4B inhibition involves: (1) a *para*-substituent that optimally engages the M pocket via a strong hydrogen bond (e.g., 4-OH) or robust hydrophobic complementarity (e.g., 4-OCH_3_); (2) favorable electronic character at the *para* position; (3) simultaneous, stable engagement of the hydrophobic P-clamp (Ile410, Phe446); and (4) proper orientation via a key hydrogen bond between the ligand carboxylate and Gln443. This integrated SAR model, grounded in multifaceted structural analysis, robustly explains the observed activity trends and provides a solid foundation for the rational design of more potent and selective PDE4B inhibitors.

### 3.6 *In Silico* ADMET Profiling of the Lead Compound

As summarized in Supporting Table S5, computational ADMET profiling of *p*-coumaric acid and *trans*-4-methoxycinnamic acid reveals a profile with several promising drug-like properties. Both compounds exhibit favorable oral absorption potential, characterized by moderate to high passive permeability and a lack of P-glycoprotein-mediated efflux, culminating in a prediction of high oral bioavailability. Furthermore, their inability to cross the blood-brain barrier minimizes the risk of CNS-related side effects for peripheral applications, and they present a low risk of cardiotoxicity (hERG) and genotoxicity (AMES).

The analysis also pinpointed several ADMET parameters that warrant attention in future optimization efforts. These include potential liabilities such as CYP2C8 inhibition-which, while posing a risk for drug–drug interactions, is a common and addressable issue in lead optimization-signals for hepatotoxicity, skin sensitization, and eye irritation, often linked to structural alerts that can be mitigated by reducing overall compound reactivity. Additionally, the suboptimal pharmacokinetic profile, indicated by a short half-life and limited tissue distribution, represents an addressable attribute rather than a critical flaw; a short half-life can be advantageous for rapid clearance, though it may require optimization for chronic indications through strategic modifications to improve metabolic stability. Critically, these identified areas provide a clear roadmap for our ongoing medicinal chemistry campaign. The scaffold’s properties are highly amenable to optimization through strategic structural modifications, such as introducing hydrophilic moieties or altering substituents on the phenyl ring to disrupt CYP2C8 binding, reduce Michael acceptor properties common to cinnamic acids, and fine-tune log *P* to improve the pharmacokinetic profile.

## 4 Conclusion

Based on the comprehensive research presented in this study, it can be concluded that monosubstituted cinnamic acid derivatives represent a promising class of PDE4B inhibitors with significant therapeutic potential for inflammatory diseases such as COPD. Our systematic evaluation identified *p*-coumaric acid (PDE4B IC_50_ = 2.2 μM) and *trans*-4-methoxycinnamic acid (PDE4B IC_50_ = 8.2 μM) as the most potent and selective leads, demonstrating robust *in vitro* inhibitory activity against PDE4B and suppression of TNF-α release in human mononuclear cells (IC_50_ = 21.5 μM and 30.8 μM, respectively), comparable to rolipram. Notably, the latter exhibited preferential selectivity for the PDE4B subtype. The *in vivo* efficacy was substantiated in LPS-induced inflammation models, where *p*-coumaric acid significantly inhibited TNF-α production (41.1%) and neutrophilia (32.5%).

The structure–activity relationship, rationalized by a robust and predictive CoMSIA model (*q* ^2^ = 0.514, *R* ^2^ = 0.971), unequivocally established that *para*-substitutions with electron-donating groups are crucial for high inhibitory activity. Integrated computational studies, combining molecular docking and molecular dynamics simulations, provided a profound mechanistic understanding of the binding mode. Our analysis revealed a dual mechanism for high-affinity binding within the M pocket: for *p*-coumaric acid, it is primarily driven by a stable hydrogen bond with His234 (96.3% occupancy), whereas for *trans*-4-methoxycinnamic acid, enhanced hydrophobic interactions with the M pocket residues compensate for the lack of this hydrogen bond. In both cases, robust hydrophobic interactions with the conserved “P-clamp” (Ile410, Phe446) are essential, with His234 identified as a major energy contributor for the hydroxy derivative. This refined model corrects the static docking pose and highlights the dynamic nature of the interaction.

Furthermore, in silico ADMET profiling predicted favorable drug-like properties for the lead compounds, including high oral bioavailability and low CNS penetration, while identifying manageable liabilities such as CYP2C8 inhibition for future optimization. In summary, this work not only identifies *p*-coumaric acid and *trans*-4-methoxycinnamic acid as valuable anti-inflammatory lead compounds but also provides a solid foundation, through well-established SAR and an elucidated binding mechanism, for the rational design and development of novel, potent, and selective PDE4B inhibitors based on the cinnamic acid scaffold.

## Supplementary Material



## Notes

Crystal structure information for cross-docking (Table S1); detailed results of cross-docking (Table S1); detailed results of 20 redocking runs (Table S3); detailed results of cinnamic acid derivatives docked to 1XN0 (Table S4); in silico ADMET properties of cinnamic acid derivatives (Table S5); overlay of the lowest energy conformations from 20 redocking runs (Figure S1); cross-docking result of rolipram from wild-type PDE4B (PDB: 1XN0) into ten other PDE4B crystal structures (Figure S2); stability analysis of the PDE4*B*/4-hydroxycinnamic acid complex from molecular dynamics simulations (Figure S3) (PDF)
