# Supplementary material for: Combining Experimental Assays and Molecular Modeling to Evaluate Monosubstituted Cinnamic Acid Derivatives as PDE4B Inhibitors
Source: ACS Omega. 2025 Dec 10;10(50):61722–35. doi: 10.1021/acsomega.5c07879 (PMC12750277; doi:10.1021/acsomega.5c07879)
Supplement: Supplementary file 1 [file ao5c07879_si_001.pdf]

Combining Experimental Assays and Molecular Modeling to Evaluate Monosubstituted Cinnamic Acid Derivatives as PDE4B Inhibitors

Dongsheng Zhao[a*§], Wendi Jia[b§], Wanyu Gong[b§], Lingling Zhang[a] and Zining Cui[b]

[a] Department of Pharmacy, Quanzhou Medical College, Quanzhou 362000, China.

[b]State Key Laboratory of Green Pesticide, Integrative Microbiology Research Centre, Guangdong Province Key Laboratory of Microbial Signals and Disease Control, College of Plant Protection, South China Agricultural University, Guangzhou 510642, China.

* E-mail addresses: 394886093@qq.com (D. Z.)

[§]These authors contributed equally to this work.

Table S1: Crystal Structure Information Used for Cross-Docking

PDB ID	Resolution(Å)	R-Value Free	Mutation	Ligand	Essential active site residues						
1XN0	2.31	0.250(Depositor), 0.249 (DCC)	N	(R,S)-Rolipram	GLN443	PHE446	ILE410	ASP392	HIS234	HIS274	ASP275
1XM6	1.92	0.227(Depositor), 0.235 (DCC)	N	(R)-Mesopram	GLN443	PHE446	ILE410	ASP392	HIS234	HIS274	ASP275
4KP6	1.50	0.198(Depositor), 0.200 (DCC)	N	[1,3,5]Triazine Derivative	GLN443	PHE446	ILE410	ASP392	HIS234	HIS274	ASP275
4MYQ	1.90	0.222(Depositor), 0.220 (DCC)	N	Pyrimidin Derivative	GLN615	PHE618	ILE582	ASP564	HIS406	HIS446	ASP447
5OHJ	1.60	0.212(Depositor), 0.220 (DCC)	N	Pyrrolidinyl Derivative	GLN615	PHE618	ILE582	ASP564	HIS406	HIS446	ASP447
2QYL	1.95	0.239(Depositor), 0.230 (DCC)	N	NPV(naphthyridin derivative)	GLN443	PHE446	ILE410	ASP392	HIS234	HIS274	ASP275
1XLX	2.19	0.282 (Depositor), 0.288 (DCC)	Y	Cilomilast	GLN443	PHE446	ILE410	ASP392	HIS234	HIS274	ASP275
1XLZ	2.06	0.237 (Depositor), 0.241 (DCC)	Y	Filaminast	GLN443	PHE446	ILE410	ASP392	HIS234	HIS274	ASP275
1XMY	2.40	0.298(Depositor), 0.295 (DCC)	Y	(R)-Rolipram	GLN443	PHE446	ILE410	ASP392	HIS234	HIS274	ASP275
3D3P	1.75	0.241 (Depositor), 0.240 (DCC)	Y	Pyrazolopyridine	GLN443	PHE446	ILE410	ASP392	HIS234	HIS274	ASP275

3O56	2.42	0.260 (Depositor), 0.262 (DCC)	Y	Pyrazolopyridine derivative	GLN443	PHE446	ILE410	ASP392	HIS234	HIS274	ASP275
Role	/	/	/	/	H-bond anchor	P clamp (upper jaw)	P clamp (lower jaw)	Zn^{2+} coordina tion	Zn^{2+} coordina tion	Zn^{2+} coordina tion	Zn^{2+}/Mg^{2+} coordinati on

Table S2: Detailed results of cross-docking.

PDB ID	Chain	Grid Center (X, Y, Z) (Å)	Grid Size (X, Y, Z) (Å)	Notes	Binding Energy (kcal/mol)	Reference RMSD(Å)*
1XN0	A	-2.521, -2.144, -16.530	70, 70, 70	Reference structure	/	/
1XM6	A	41.810, -2.215, -16.307	70, 70, 70	Derived from 1XN0 Gln443	-9.34	1.45
4KP6	A	-33.035, 91.753, 110.980	70, 70, 70	Derived from 1XN0 Gln443	-8.80	0.58
4MYQ	A	-23.171, 30.403, -10.770	70, 70, 70	Derived from 1XN0 Gln443	-8.54	0.88
5OHJ	A	-34.226, -13.749, 32.266	70, 70, 70	Derived from 1XN0 Gln443	-7.79	1.48
2QYL	A	33.891, 39.286, 4.167	70, 70, 70	Derived from 1XN0 Gln443	-8.22	0.94
1XLX	A	-2.614, -2.211, -16.678	70, 70, 70	Derived from 1XN0 Gln443	-8.37	1.46
1XLZ	A	-2.583, -2.166, -16.505	70, 70, 70	Derived from 1XN0 Gln443	-8.22	1.46
1XMY	A	47.420, -2.264, 69.813	70, 70, 70	Derived from 1XN0 Gln443	-8.07	0.54
3D3P	A	41.648, 50.104, 70.555	70, 70, 70	Derived from 1XN0 Gln443	-8.23	0.85
3O56	A	41.685, 50.148, 70.197	70, 70, 70	Derived from 1XN0 Gln443	-8.13	0.92
Mean ±SD	/	/	/	/	-8.37 ± 0.44	1.06 ± 0.37

* RMSD was calculated between the docked pose and the pose of the same ligand from the PDB structure 1XN0..

Table S3: Detailed results of 20 re-dock runs.

Run ID	Lowest Binding Energy (kcal/mol)	RMSD of Best Pose	Total Clusters	Multi-member Clusters	Largest Cluster Size	Largest Cluster Avg Energy (kcal/mol)	2nd Largest Cluster Size	2nd Largest Cluster Avg Energy (kcal/mol)	3rd Largest Cluster Size	3rd Largest Cluster Avg Energy (kcal/mol)	% in Top Cluster
01	-8.03	1.56	8	4	36	-7.81	4	-7.89	4	-7.17	72
02	-8.03	1.46	6	6	23	-7.84	18	-7.75	5	-6.97	46
03	-8.01	1.41	9	5	23	-7.75	15	-7.76	3	-7.30	46
04	-7.97	0.42	4	3	37	-7.75	7	-7.75	5	-7.31	74
05	-7.99	1.46	5	5	22	-7.81	20	-7.76	4	-7.49	44
06	-8.05	1.46	5	4	21	-7.66	18	-7.75	6	-7.14	42
07	-7.99	1.47	5	4	36	-7.75	6	-7.39	5	-7.06	72
08	-8.05	1.53	6	5	19	-7.68	16	-7.80	7	-7.23	38
09	-7.94	1.47	8	3	40	-7.74	3	-7.42	2	-7.16	80
10	-7.98	1.44	8	5	23	-7.83	15	-7.66	4	-6.84	46
11	-8.00	1.49	7	4	27	-7.79	14	-7.73	4	-6.93	54
12	-8.04	0.88	8	4	26	-7.80	13	-7.76	5	-7.03	52
13	-8.01	1.42	5	3	43	-7.79	3	-6.76	2	-7.27	86
14	-8.06	1.43	6	4	24	-7.79	17	-7.69	4	-7.05	48
15	-8.01	1.46	5	3	29	-7.76	12	-7.58	7	-7.22	58
16	-7.92	0.48	8	4	37	-7.72	5	-7.50	2	-6.97	74
17	-8.00	1.48	7	4	27	-7.80	13	-7.73	4	-7.17	54
18	-7.99	1.50	7	4	36	-7.79	5	-7.15	4	-7.13	72

19	-7.98	1.48	5	4	41	-7.74	4	-6.94	2	-7.94	82
20	-8.01	1.42	6	4	38	-7.77	5	-7.36	3	-6.98	76
Mean ±SD	-8 ± 0.04	1.34 ± 0.33	6.4 ± 1.43	4.1 ± 0.79	30.4 ± 7.75	-7.77 ± 0.05	10.65 ± 5.91	-7.56 ± 0.3	4.1 ± 1.52	-7.17 ± 0.24	60.8 ± 15.5

Table S4: Detailed results of Cinnamic Acid Derivatives docked to 1XN0

Compound	Name	N (non-hydrogen heavy atoms)	IC_{50}(uM)	pIC_{50}	Binding Energy (Kcal/mol)	Total Clusters	Multi-member Clusters	Largest Cluster Size	Largest Cluster Avg Energy (kcal/mol)	% in Top Cluster	Ligand Efficiency
1	*trans*-cinnamic acid	11	90.3	-1.96	-4.09	5	5	35	-3.94	70	-0.37

2	*ortho*-coumaric acid	12	41.4	-1.62	-4.62	5	2	47	-4.55	94	-0.39
3	*meta*-coumaric acid	12	28.4	-1.45	-4.85	7	6	25	-4.19	50	-0.40
4	*p*-coumaric acid	12	2.2	-0.34	-4.31	7	5	28	-4.23	56	-0.36
5	*trans*-2-methoxycinnamic acid	13	51.1	-1.71	-4.41	5	5	33	-4.62	66	-0.34
6	*trans*-3-methoxycinnamic acid	13	32.8	-1.52	-4.64	9	4	22	-4.04	44	-0.36
7	*trans*-4-methoxycinnamic acid	13	8.2	-0.91	-4.53	5	4	31	-4.42	62	-0.35
8	*trans*-2-methylcinnamic acid	12	136	-2.13	-4.51	5	4	42	-4.26	84	-0.38
9	*trans*-3-methylcinnamic acid	12	102	-2.01	-4.62	4	3	29	-4.20	58	-0.39
10	*trans*-4-methylcinnamic acid	12	85	-1.93	-4.48	6	6	25	-4.36	50	-0.37
11	*trans*-2-chlorocinnamic acid	12	82	-1.91	-4.79	5	5	33	-4.30	66	-0.40
12	*trans*-3-chlorocinnamic acid	12	63	-1.8	-4.87	5	5	23	-4.28	46	-0.41
13	*trans*-4-chlorocinnamic acid	12	44	-1.64	-4.73	4	3	22	-4.59	44	-0.39
14	*trans*-4-nitrocinnamic acid	14	111	-1.99	-3.80	3	3	31	-3.40	62	-0.27
15	*trans*-4-formylcinnamic acid	13	101	-2.00	-4.85	5	3	32	-4.59	64	-0.37
16	*trans*-4-fluorocinnamic	12	33	-1.52	-3.97	6	5	21	-3.87	42	-0.33

	acid										
17	*trans*-4-bromocinnamic acid	12	87	-1.94	-5.06	4	3	28	-4.87	56	-0.42
18	*trans*-4-dimethylaminocinnamic acid	14	33	-1.52	-4.69	5	5	17	-3.86	34	-0.34
19	*trans*-4-trifluoromethylcinnamic acid	15	97	-2.05	-4.43	4	3	20	-4.28	40	-0.30
/	rolipram	20	2	-0.30	-8.03	8	4	36	−7. 81	72	-0.40

Table S5. *In silico* ADMET properties of cinnamic acid derivatives*

Property Category	Parameter	*p*-Coumaric Acid		*trans*-4-Methoxycinnamic Acid	
		Predicted Value	Risk Level	Predicted Value	Risk Level
Absorption	PAMPA	++	Low	+	Low
	Pgp-substrate	---	Low	---	Low
	F20%	+++	Low	+++	Low
Distribution	BBB	---	Low (for non-CNS drugs)	---	Low (for non-CNS drugs)
	PPB	68.3%	Moderate	76.7%	Moderate
	VDss (L/kg)	0.202	Moderate	0.182	Moderate
Metabolism	CYP3A4 substrate	---	Low	---	Low
	CYP2C8 inhibitor	+++	High	+++	High
Excretion	T~1/2~ (h)	1.57	High	1.41	High

Property Category	Parameter	*p*-Coumaric Acid		*trans*-4-Methoxycinnamic Acid	
		Predicted Value	Risk Level	Predicted Value	Risk Level
Toxicity	hERG Blockers	0.049	Low	0.063	Low
	Human Hepatotoxicity	0.78	High	0.757	High
	Skin Sensitization	0.783	High	0.632	High
	Eye Irritation	0.999	High	0.997	High
	AMES Toxicity	0.219	Low	0.279	Low

* For the specific definitions of the parameters, please refer to the paper.

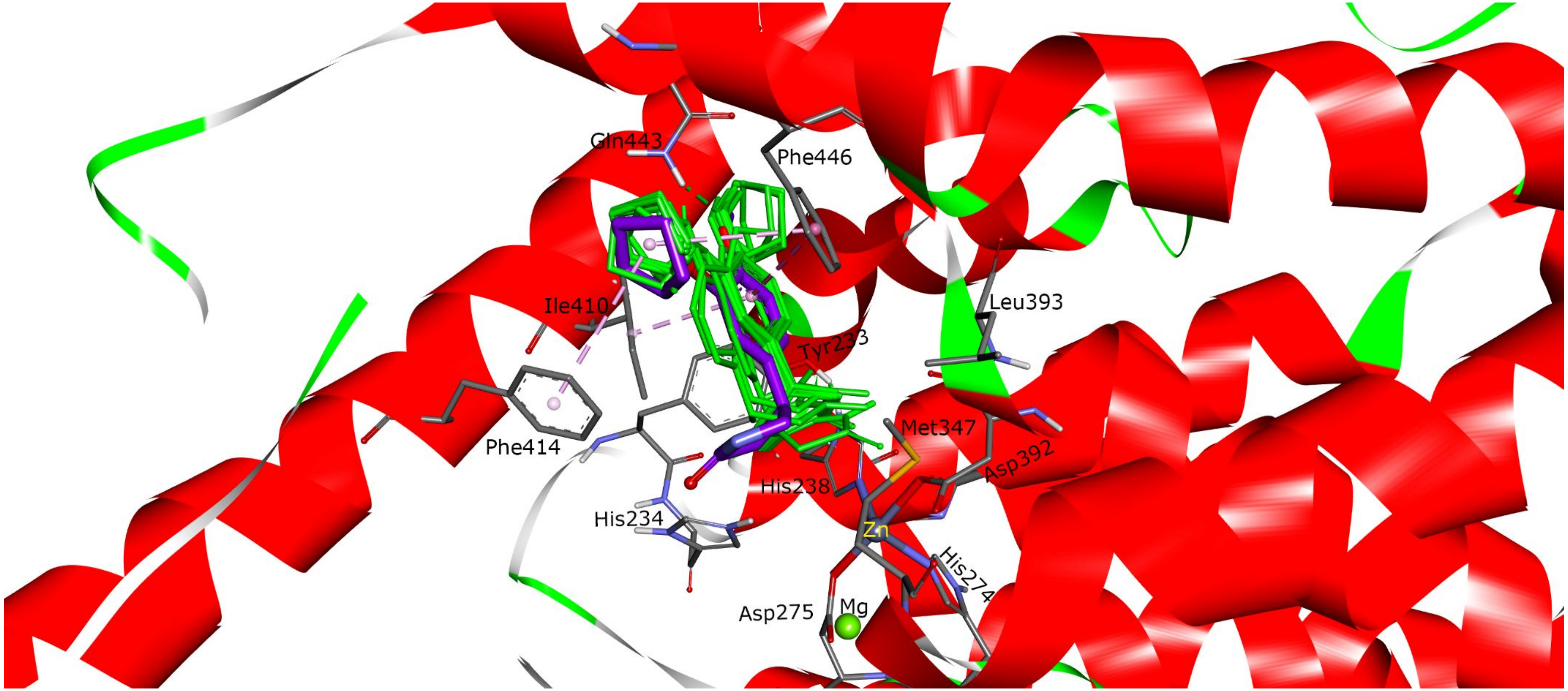

Figure S1. Overlay of the lowest energy conformations from 20 re-docking runs.

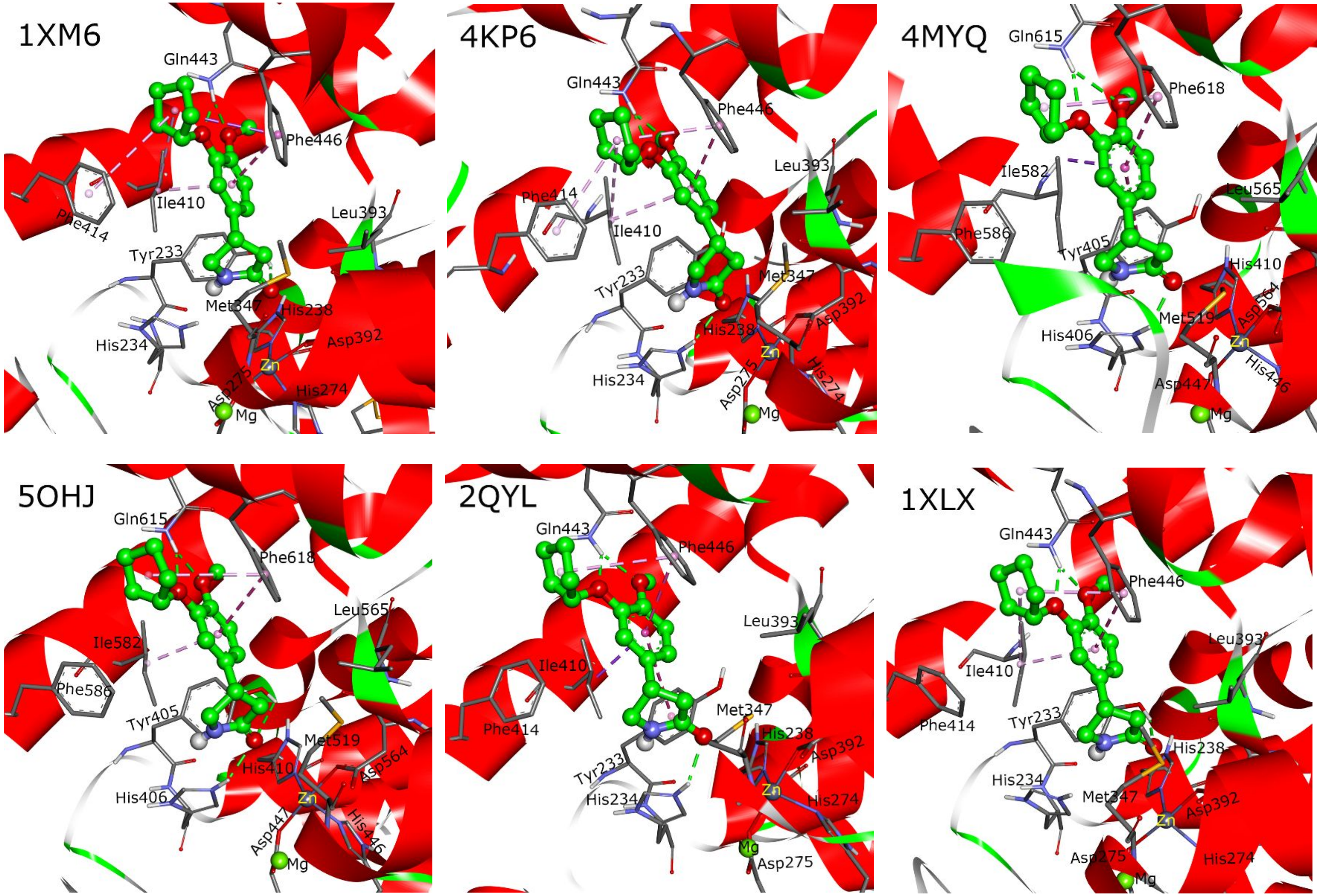
1XM6
Gln443
Phe446
Ile410
Phe414
Leu393
Tyr233
Met347
His238
Asp392
His234
Asp275
Zn
His274
Mg
4KP6
Gln443
Phe446
Leu393
Phe414
Ile410
Tyr233
Met347
His238
Asp392
His234
Zn
Asp275
His274
Mg
4MYQ
Gln615
Phe618
Ile582
Leu565
Phe586
Tyr405
His410
Met519
Asp564
His406
Zn
Asp447
His446
Mg
5OHJ
Gln615
Phe618
Leu565
Ile582
Phe586
Tyr405
Met519
His410
Asp564
His406
Zn
Asp447
His446
Mg
2QYL
Gln443
Phe446
Leu393
Ile410
Phe414
Met347
His238
Asp392
Tyr233
His234
Zn
His274
Mg
Asp275
1XLX
Gln443
Phe446
Leu393
Ile410
Phe414
Tyr233
His238
His234
Met347
Asp392
Zn
Asp275
His274
Mg

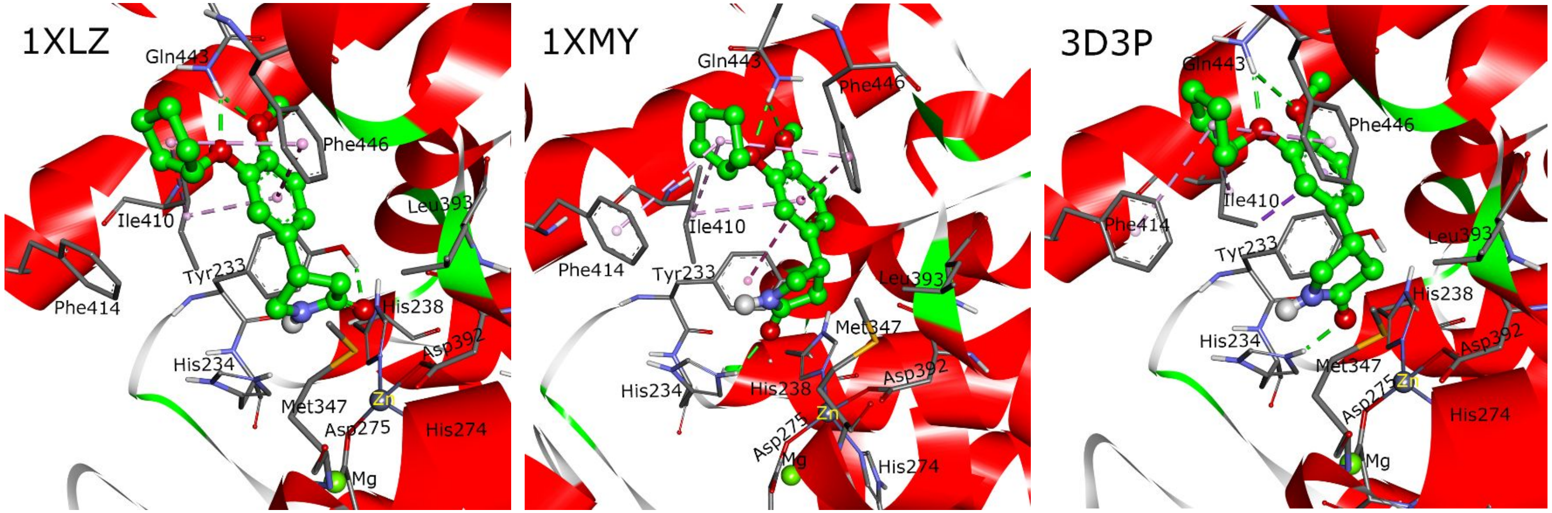

Figure S2. Cross-docking result of rolipram from the wild-type PDE4B (PDB: 1XN0) into ten other PDE4B crystal structures (with 3O56 already depicted in the paper).

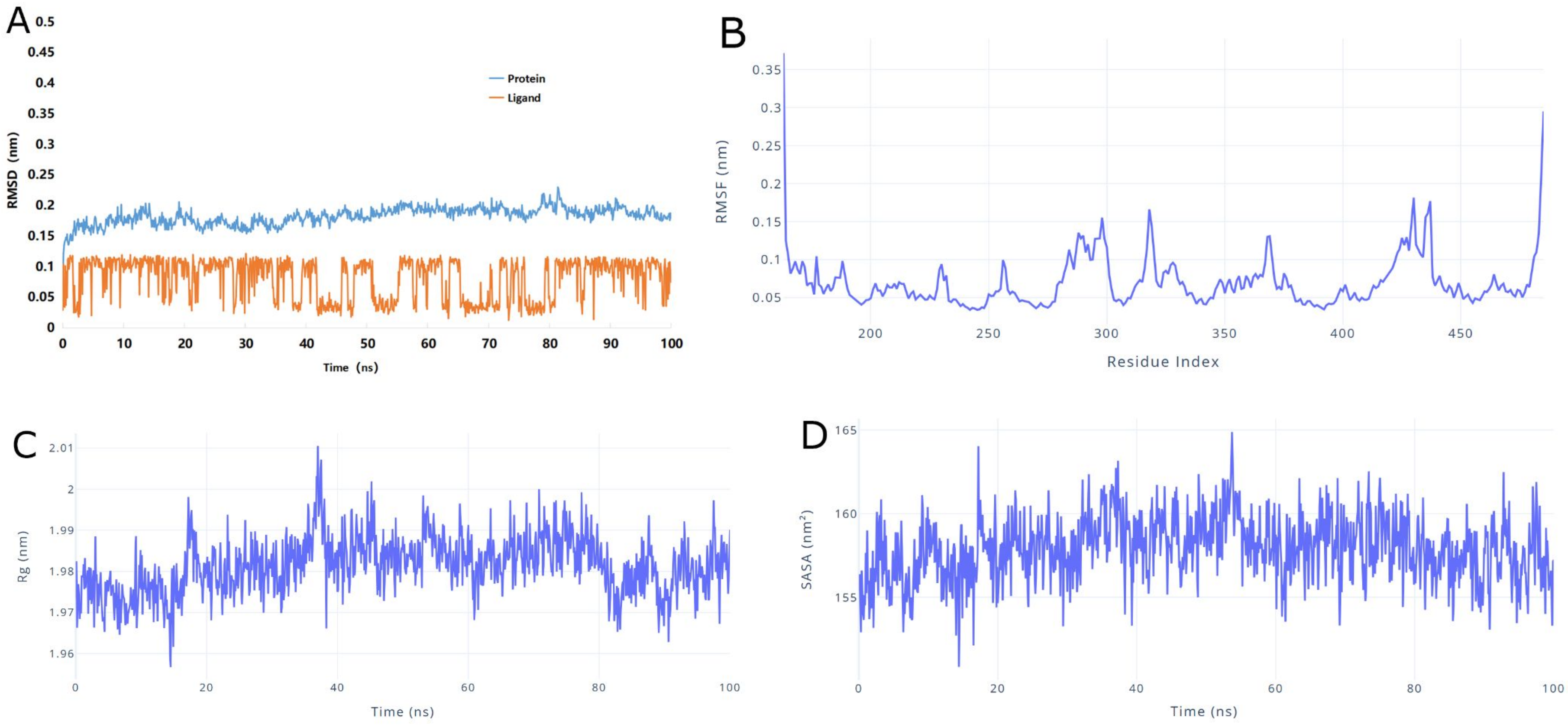
A
Protein
Ligand
RMSD (nm)
Time (ns)
B
RMSF (nm)
Residue Index
C
Rg (nm)
Time (ns)
D
SASA (nm²)
Time (ns)

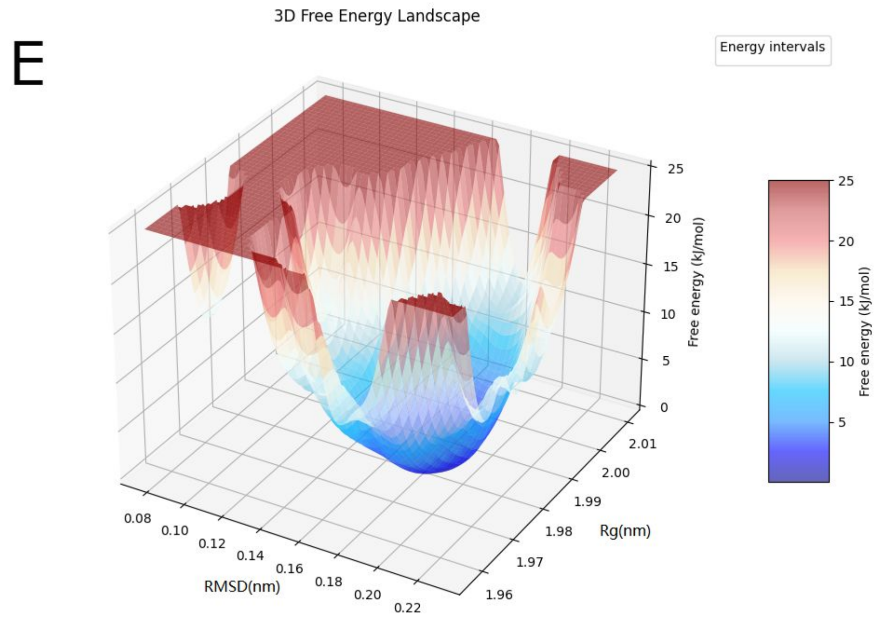

Figure S3. Stability analysis of the PDE4B/4-hydroxycinnamic acid complex from molecular dynamics simulations. (A) Root-mean-square deviation (RMSD) of the protein backbone. (B) Root-mean-square fluctuation (RMSF) of protein residues. (C) Radius of gyration (Rg). (D) Solvent-accessible surface area (SASA). (E) Free energy landscape (FEL) projected onto the RMSD and Rg.
